# Linking environmental psychology and critical social psychology: Theoretical considerations toward a comprehensive research agenda

**DOI:** 10.3389/fpsyg.2022.947243

**Published:** 2022-09-02

**Authors:** Thomas Kühn, Sebastian Bobeth

**Affiliations:** Chair of Work and Organizational Psychology/Erich Fromm Study Center, International Psychoanalytic University Berlin, Berlin, Germany

**Keywords:** critical social psychology, critical environmental psychology, pro-environmental behavior, qualitative research, life course research

## Abstract

In order to foster pro-environmental behavior in the midst of a global ecological crisis, current research in environmental psychology is often limited to individual-related factors and theories about conscious processing. However, in recent years, we observe a certain discontentment with the limitations of this approach within the community as well as increasing efforts toward broadening the scope (e.g., promotions of collective and social identity processes). In our work, we aim for a closer investigation of the relations between individuals, societal factors, and pro-environmental actions while considering the role of the unconscious. We hereby draw on the work of critical social psychology (CSP). From a life course perspective, we emphasize the important role of socialization, institutional and cultural contexts for mindsets and related perceptions, decisions and actions. This link between the individual and the society enables us to understand biographical trajectories and related ideologies dominant within a society. We seek to show that the approach of CSP is helpful for understanding why efforts of establishing pro-environmental actions on a large scale are still failing. In this article, we discuss the theoretical links between environmental psychology and CSP as well as possible implications, paving the way for a comprehensive future research agenda.

## Introduction

Being acknowledged as a sub-discipline of psychology from the 1960s onward, environmental psychology has been concerned with the interplay of humans and their environment. An early focus that is still an important part of environmental psychology today is the question of how various (built, natural, social, today also digital) environments influence perceptions, cognitions, feelings, and behavior of humans. Furthermore, environmental psychology has a tradition of engaging with questions of environmental sustainability^1^ since the emergence of larger-scale awareness of ecological crises in science and society in the 1970s and 1980s (for more details on main characteristics and the historical development of the field, see, e.g., Gifford, 2007, pp. 199–207; Pol, 2006, 2007; Steg et al., 2013, pp. 2–6). Since the beginning of the 21st century, environmental psychologists have been particularly concerned with the link between human behavior and the anthropogenic destabilization and destruction of global ecosystems, most prominently in the case of anthropogenic climate change. Recognizing climate change as a major threat to the foundations of human existence (Intergovernmental Panel on Climate Change, 2022a, pp. 10–21), environmental psychologists investigate the underlying reasons for environmentally significant human behavior, the potential for behavior change, as well as concrete measures and interventions to foster behavior change. The unifying objective of theoretical and practical efforts in the field is that of ecological, social, and economic sustainability (Steg et al., 2013, p. 4).

Undoubtedly, environmental psychology has advanced our understanding of factors that influence environmentally significant human behavior. A rich body of studies shows a multitude of factors both within individuals (such as attitudes, control beliefs, values and moral norms, affects, personality factors) and outside individuals (such as social norms, costs in terms of time, effort, and money, aspects of infrastructural design, policy measures) that influence environmentally significant behavior (e.g., Bamberg and Möser, 2007; Klöckner, 2013; Gifford and Nilsson, 2014; van der Linden and Goldberg, 2020). While, in general, diversity of theories and research methods is one of the characteristics of the field (Steg et al., 2013, p. 6; for an overview of common methods, see Gifford, 2016), research in this tradition is often based on action models, quantitative survey studies, and correlational analyses of possible underlying factors of the behaviors of interest. Based on such theories and respective empirical findings, environmental psychologists traditionally suggest leverage points to promote behavior change to policy makers (e.g., Gardner and Stern, 2009; Wolske and Stern, 2018; Nielsen et al., 2020). Moreover, environmental psychologists have a long tradition of directly implementing and scientifically evaluating concrete measures to promote behavior change in practice, linking with insights from action research (e.g., McKenzie-Mohr, 2000; Abrahamse et al., 2005; Steg and Vlek, 2009; Osbaldiston and Schott, 2012; Schultz, 2014).

Despite these achievements, our observation from being engaged in the environmental psychology community is also that of a certain level of discontentment, reflecting current challenges of the field. One major challenge for researchers working on topics related to climate change is their high level of knowledge about the tremendous dangers of climate change and about various possible actions toward mitigation while simultaneously observing that humanity is still failing to address the climate crisis appropriately (*implementation gap*; Intergovernmental Panel on Climate Change, 2022b, p. 15). Continually experiencing this contrast can be a driver, but also a heavy mental burden that can be experienced as overwhelming and can lead to severe negative effects on mental health (e.g., Head and Harada, 2017; Clayton, 2018). More specifically related to the field of environmental psychology, there seems to be a growing dissatisfaction of the lack of impact that research has so far made in practice, leading to concerns that “psychology is falling short of its potential” (Nielsen et al., 2020, pp. 3–4). Searching for explanations, environmental psychologists recently have been questioning the primary focus on individual (consumption) behavior change in the discipline (e.g., Amel et al., 2017, p. 3; Wallis et al., 2021, pp. 184–186; Wullenkord and Hamann, 2021, pp. 1–2) and the spheres of behavior typically addressed^2^ (e.g., Moser and Kleinhückelkotten, 2018, pp. 627–631; Nielsen et al., 2020, pp. 3–6). Some alternative suggestions researchers have recently made are that environmental psychologists should focus more on predictors of high-impact behaviors (e.g., Wolske and Stern, 2018; Nielsen et al., 2020, 2021), on predictors of collective action, social and societal change, and change of cultural norms (e.g., Amel et al., 2017; Fritsche et al., 2017; Wallis et al., 2021), on more inter- and trans-disciplinary collaborations (Clayton et al., 2016a), or on the links between individual behavior change and changes in the socio-economic system (e.g., Wallis et al., 2021; Wullenkord and Hamann, 2021).

Some researchers from the discipline go a step further when investigating challenges of the discipline, being more fundamental in their critique. As the current ecological crisis is rooted in the economic systems, lifestyles, and ideologies in the industrialized nations and these strongly influence the academic careers and practices of academic research, they suggest that environmental psychology would benefit from a stronger reflection of ideological influences as well as influences of structures and power when trying to play an active role in socio-ecological change (Uzzell and Räthzel, 2009; Krenzer and Kreil, 2019; Räthzel and Uzzell, 2019; Schmitt et al., 2020; Krenzer, 2020; Adams, 2021). Consequently, environmental psychology’s key assumptions, paradigms, and methods would have to be questioned and readjusted to conduct more meaningful and practically relevant research. One major criticism in this respect is that theories and research practice in the field have not only been focusing too much on individual behavior change, but have been treating individuals as privatized, static, egocentric, and more or less independent actors (e.g., Räthzel and Uzzell, 2019, pp. 1377–1380; Schmitt et al., 2020, pp. 124–130; Adams, 2021, pp. 14–15). This concept of human beings would downplay the importance of social relations and the larger social context, societal structures and power relations, as well as the dynamics in individual biographies and societal processes (Ibid.). For example, in this view, it would be of much importance what kinds of pro-environmental attitudes, values, or social norms are (re)produced in the concrete context of individuals and how these are “shaped through relations of power, the economic structure of society, and the dominant ideologies and forms of politics” (Räthzel and Uzzell, 2019, p. 1390). Investigations of the social embeddedness of intra-individual processes would have to be sensitive for the complexity, ambivalences, contradictions, and processual nature of everyday life as well as the complex role of emotions and affects. This challenges the discipline’s predominant methodological approach of quantitative analyses with a focus on the *status quo* in the present (or a relative short timeframe, respectively; examples would be correlational studies or laboratory and field experiments; see above, cf. Lertzman, 2019, pp. 26–28). However, by critically challenging the *status quo*, environmental psychologists would have to take political stances more explicitly and be more directly involved in ongoing processes of change, which might conflict with the ideal of political neutrality prevalent in the discipline and in science in general (Krenzer and Kreil, 2019, pp. 167–168).

To sum up, despite undeniable achievements of the past, we are currently observing a discipline with researchers dissatisfied on different levels. The central challenge seems to be how the discipline can adapt to increase its impact and take on a more central role in promoting large-scale societal and systemic change within the social-ecological transformation. In this respect, we observe a general openness toward new directions and more multiperspectivity in the community.

For such an undertaking, we argue that it is both fruitful and necessary to engage with the aforementioned issues that critical researchers have pointed out, paving the way toward a *critical environmental psychology*.^3^ In the following, we will outline how, in this respect, the perspective of *critical social psychology* could be combined with questions of and challenges within environmental psychology. Especially from the view of a life course perspective (Kühn, 2015), our objective is to introduce pillars of a suitable framework and demonstrate possibilities for its application in order to spark further discussions.

In section “Alternative trends in current environmental psychology research fostering a critical perspective,” we point out developments in environmental psychology that, from our perspective, link well with the aforementioned approach. In section “Fundamental considerations on the importance of critical social psychology for environmental psychology,” we substantiate and elaborate this perspective by offering fundamental considerations on the benefits of critical social psychology for environmental psychology. In section “Critical environmental psychology from a life course perspective,” we demonstrate the potential for future research by providing examples of a qualitative approach within life course research. Finally, in section “Conclusion,” we conclude and provide an outlook for further research.

## Alternative trends in current environmental psychology research fostering a critical perspective

Since its origins, research in the field of environmental psychology has always been diverse, integrating ideas from and orienting toward other scientific disciplines and being constantly influenced by new scientific knowledge from other fields as well as emerging trends and practices in the society and macro-developments (Steg et al., 2013, pp. 5–6). Here, we will briefly describe some theories and trends we have observed in recent years that, from our point of view, are challenging or enhancing the predominant course of research in the field. The collection is not intended to be exhaustive. The approaches that we mention are investigating the interplay between humans and their environment while integrating aspects of the societal context, social inequalities, social change, or social discourse, dealing with questions of inequality or embedding non-intentional or non-rational processes.

Such approaches are usually implemented by specific working groups, within (time-limited) projects or by researchers of a similar tradition or orientation, sometimes within research clusters. While they are certainly known by many researchers in the field of environmental psychology, the ideas and results have, at least so far, not been integrated systematically in the quantitative individual-focused research based on action models that has dominated the field in its recent history. Furthermore, the researchers investigating these perspectives rarely systematically linked the mentioned approaches to one another. By this, we are in no way calling for the development of a coherent, comprehensive framework for the discipline, which might prove impossible due to the variety and complexity of the topics of interest (Stern, 2000). However, as stated before, we are emphasizing that research in the discipline should systematically implement perspectives from a fundamentally critical stance. In this respect, from our point of view, these trends and theories accompany, enhance, and or pave the way toward a critical environmental psychology.

One of such trends is the increased interest in understanding and promoting social movements. The interactions of humans with their social environment has been of interest to the discipline from the beginning (Pol, 2006, p. 97). Furthermore, the relevance of public-sphere behavior and activism have always been somewhat on the radar of environmental psychologists concerned with ecological crises (e.g., Stern, 2000). In the last decade or so, environmental movements and collective forms of protest against ineffective large-scale political actions to mitigate climate change (such as the *Fridays for Future* movement) have grown, gained more and more media attention, and increased the pressure on politicians. Better understanding people’s motivation to engage with, stay active in, and promote social movements currently has gained a lot of momentum in the environmental psychologists’ community (e.g., Wallis et al., 2021). For example, researchers have been applying *social identity theory* (Tajfel and Turner, 1979) and the *social identity model of collective action* (van Zomeren et al., 2008) to explain engagement in environmental movements (Fritsche et al., 2017). Challenging the dominance of research on private-sphere individual behavior, looking at collective phenomena and collective behavior seems a promising path regarding that the magnitude of challenges and solutions has to be addressed at the societal level (Amel et al., 2017). In terms of critical agenda setting, increasingly focusing on perceived (social) injustice, social conflicts, and related emotions are noteworthy contributions. Furthermore, a stronger emphasis on social movements and social change can contribute to challenging predominant structures of social and environmental injustice.

Another noteworthy trend is that some environmental psychologists are currently striving for strengthening the links between their discipline and perspectives from (sociotechnical) system transformation research. One framework from system transformation research that is helpful to understanding the interplay between small-scale/individual activities and macro factors in social change is the *multi level perspective* (Geels, 2004; Geels and Schot, 2007). The theory describes how the *status quo* is manifested as a result of the complex interplay, interdependencies and path dependencies within and between industry, policy making, industry, technology, culture, and science. Furthermore, it offers perspectives on how large-scale societal change can take place (e.g., how pro-environmental alternatives to current practices and lifestyles can become mainstream). Environmental psychologists are currently striving to integrate theories from their field with this perspective to better understand how individual behavior change is embedded in large-scale societal transformation processes (e.g., Wallis et al., 2021; Wullenkord and Hamann, 2021). The benefit of this undertaking from a critical standpoint is that important parameters of the socio-economic and societal system are no longer neglected, but systematically linked with and incorporated into research on individual cognitions and behavior. Furthermore, the implication of this link is to investigate determinants of societal and systemic change, which is needed to appropriately tackle climate change and other ecological and social problems (see section “Introduction”).

Another fruitful line of research in the discipline is linking psychological perspectives to interdisciplinary discourses on environmental justice (see, e.g., Mohai et al., 2009; Walker, 2012; Baasch, 2020) and energy justice (see, e.g., Sovacool and Dworkin, 2015; Jenkins et al., 2016, 2021). Environmental psychologists have investigated, for example, (a) subjective conceptualizations of justice related to environmental or energy-related problems or conflicts, (b) experiences and behaviors of relatively more and relatively less affected people and communities in this respect, both on the local (e.g., air, chemical, or noise pollution) and the global level (impacts of the destruction of large-scale eco-systems and, particularly, climate change), (c) the role of perceived (in)justice for pro-environmental behavior, and (d) psychological contributions to environmental conflict resolution (e.g., participation processes, mediation; for overviews of activities in the field, see, e.g., Clayton et al., 2016b; Kals and Baier, 2017; Baasch, 2020). While incorporating and highlighting societal issues of injustice from the very beginning, research from this perspective has recently become even more plural (Kals and Baier, 2017, pp. 82-88) by also investigating questions of social (in)justice as well as more intersectional and critical perspectives and emphasizing the role of emotions and concepts such as discourse and agency to explain and foster resistance, participation, and change (e.g., Manning and Amel, 2014; Baasch, 2020; Cunsolo et al., 2020; Fernandes-Jesus et al., 2020; Groves et al., 2021; Makovi and Kasak-Gliboff, 2021; Nguyen and Batel, 2021). From a critical perspective, such studies are clearly important as they recognize the importance of predominant structures, power issues, and social injustices. Moreover, they investigate how such issues and discourses are perceived, reconstructed, and linked to environment-related cognitions and behavior. Research of this tradition is shedding light on boundaries set by systemic factors, thereby highlighting the limitations of individual behavior change and the need for structural and political action, but also helps to identify and bring forth new forms of agency.

When considering environmental justice, it is crucial to not only look at research and approaches from the Global North, but to systematically include approaches from the Global South (e.g., Kühn and Souza, 2006; Rehbein, 2011). Jodhka et al. (2017) show how dangerous a one-sided view of social environments can be. For example, it is especially problematic when development processes of societies are linked to explicit or implicit basic assumptions of modernization that are based exclusively on concepts of the Global North. From such a perspective, history appears as “an evolution toward a superior model of society embodied by European and North American nation-states” (Jodhka et al., 2017, p. 2). Jodhka et al. argue that “these assumptions contribute to the resilience of inequality and need to be overcome” (Ibid.). Important critical research and agenda setting has been done by researchers from environmental psychology from the Global South (e.g., with regard to environmental planning and urban design, people-place-relations, and community participation, among others; Wiesenfeld and Sánchez, 2002; Wiesenfeld, 2005; Farias and Diniz, 2018; Diniz et al., 2020). Furthermore, some fruitful work including critical perspectives has emerged from dialogue between researchers from the Global North and Global South (e.g., Devine-Wright et al., 2020; Raymond et al., 2021).

Another promising line of research not systematically integrated in mainstream research in environmental psychology is the tradition of *climate psychology* (e.g., Weintrobe, 2012; Hoggett, 2019). This line of research tries to better understand the, on a large scale, inappropriate reaction of humans toward the imminent threat of climate change by drawing strongly on psychodynamic theories (Searles, 1972), emphasizing the importance of emotions, emotional work, and defense mechanisms as well as dealing with psychological phenomena such as denial, anxiety, grief, or trauma (e.g., Weintrobe, 2012; Orange, 2016; Hoggett, 2019; Dodds, 2021). Insights from this tradition have recently been linked with more traditional research approaches from environmental psychology on climate change denial and climate anxiety (e.g., Clayton and Karazsia, 2020; Wullenkord et al., 2021; Wullenkord and Reese, 2021). From a critical perspective, involving psychodynamic approaches and, thereby, emphasizing emotions and defense mechanisms, certainly adds another important dimension to a field mostly focusing on consciousness in its research (methods), i.e., cognitions, thoughts, and rationality. This improves our understanding of important roots of everyday meaning and individual perceptions of environment-related matters.

Finally, we would like to mention the work that has been done with reference to social representation theory (Moscovici, 1961; Marková, 2008). Social representation theory hypothesizes that when knowledge is produced and shared, it is always shaped by processes of gaining social and cultural meaning. I.e., knowledge would be the product of social interaction and, at the same time, individual representation (Marková, 2008). Climate change would, in most cases (in industrialized nations), not directly and consciously be experienced through the human senses, but would rather be a socially constructed concept (e.g., Moloney et al., 2014). Research in environmental psychology could benefit from integrating social representations theory to address common problems of varying understandings and definitions of key concepts among the scientific community, in the general population, and in the dialogue between both. This could also potentially strengthen the link between measurement constructs representing environmentally significant attitudes and norms, on the one side, and environmentally significant behavior, on the other side, by highlighting misconceptions, ambivalences, and uncertainties currently neglected in many studies in the field (Castro, 2006; Castro et al., 2009; Batel and Devine-Wright, 2015; Batel and Castro, 2018). The critical implication of this line of research is that it recognizes the importance of individual everyday representations of relevant concepts as well as the importance of social discourse and everyday social interaction for environment-related cognitions and behavior. This perspective has important implications in terms of critical theory building but also methodology (see section “Fundamental considerations on the importance of critical social psychology for environmental psychology”).

To sum up, researchers with ties to environmental psychology have done much fruitful work in terms of critical agenda setting and conducting critical research. However, from our point of view, the critical foundations in many of the aforementioned lines of research as well as interlinkages could be further strengthened. For example, while important critical research is being done by investigating the interdependency between individual, social, and systemic developments in several lines of research, most investigations still tend to focus on momentary captures of psychological concepts. As the relation between individuals, societal and socio-economic structures, and respective change processes is highly dynamic, stronger investigating dynamic processes in the biographies of individuals and their respective relations to their social and structural environments could further improve the understanding of this complex interplay. For this, investigating drivers of resistance and biographical fractions could be of specific interest. In this regard, researchers investigating psychodynamic mechanisms and social representations already provide fruitful insights. However, these research traditions could give even more weight to systematic investigations of (perceptions of) structural conditions and social critique. In the following section, we would like to take a step back and reflect upon how basic principles from critical social psychology could help linking and, partly, enhancing the critical work being done in the field.

## Fundamental considerations on the importance of critical social psychology for environmental psychology

The studies listed in the previous section all have quite different theoretical foundations. Not all of them define themselves as critical, even though they broaden the spectrum of research in environmental psychology, in particular by also linking societal developments and social groups to individual behavior. In order to sharpen the notion of a “critical” approach in environmental psychology, we attempt to condense this rather broad understanding into three theses.

### Thesis 1: There is a need for reflections on the image of humankind and the relationship between humans and the environment

Human behavior can be approached from a scientific perspective in different ways, for instance by focusing on attitudes, on ways of reacting to stimuli, or on different forms of social identity or social practice construction (Batel et al., 2016). This always implicitly implies a certain image of humankind and of the integration of people into society, even if this is not always reflected. In this context, especially with regard to numerous experimental designs, critical scholars point out the dangers of reductionist approaches that try to capture relationships in tangible causal models but fail to acknowledge the complexity of human action. When one focuses only on what can be clearly observed and measured, one quickly paints a distorted picture of social reality.

To illustrate this, let us refer to a quote by Fromm (1976, 1997, originally published in 1976) from the book *To Have or To Be*:

The first requirement in the possible creation of the new society is to be aware of the almost insurmountable difficulties that such an attempt must face. The dim awareness of this difficulty is probably one of the main reasons that so little effort is made to make the necessary changes. Many think: ‘Why strive for the impossible? Let us rather act as if the course we are steering will lead us to the place of safety and happiness that our maps indicate.’ Those who unconsciously despair yet put on the mask of optimism are not necessarily wise. But those who have not given up hope can succeed only if they are hardheaded realists, shed all illusions, and fully appreciate the difficulties. This sobriety marks the distinction between awake and dreaming ‘utopians’ (Fromm, 1997, p. 141).

Fromm distinguishes here between a displayed optimism and an unconscious despair. This is based on a psychodynamically founded view of human beings, according to which we are not aware of certain feelings and drives of our actions and cannot easily become aware of them. From this perspective, optimistic attitudes that might be elicited from surveys using questionnaires can be understood as a mask and, at a deeper level, as an expression of hopelessness.

Regardless of whether and to what extent we are willing to follow Fromm’s train of thought at this point, it becomes clear: Human perception and action cannot be understood independently of the image one forms of it – and this means that, also for environmental psychology, a reflection on both psychological and social-theoretical basic assumptions is required in order not to argue in a (post-)positivistic way. This includes reflections on what constitutes the environment, e.g., sense-making processes of the concept of *nature* (Castree, 2013) as well as consequences for political ecology and environmental governmentality (Luke, 2016).

### Thesis 2: Human perception and action are to be understood from a dynamic perspective, which takes into account historical-cultural contexts, biographical socialization processes and the connection between past, present and future in the form of different narratives

None of us is timeless or spaceless. We are all bound up in certain historical contexts and societies with certain culturally shaped patterns of imagination. Even as scientists we cannot free ourselves from this. This also applies to our personal development. We have become who we are today within the framework of a long-lasting biographical development, which, on the one hand, is connected with biological maturation and aging processes and, on the other hand, is to be understood as the result of socialization processes teaching us ideas of good and bad, right and wrong, etc.

Therefore, it is not only important for scientists in psychology and humanities to always reflexively question their own image of human beings and their social integration, but also to deal with how the people in the focus of an investigation see themselves and the world. How we perceive and act always depends on the subjective interpretation of our environment. Hence, people who are in the focus of investigations must not appear only as placeholders or as projection surfaces for assumptions of the scientists. However, the empirical examination of life trajectories, biographical decisions, and different basic assumptions in the population often comes up short. In this sense, Honneth (2007, p. 49) criticizes that the question of the motivational constitution of subjects should actually be at the center of sociocritical debate, but this is hardly the case in any approach in the interdisciplinary field of social criticism. Instead, it is mostly considered sufficient to expose grievances in society with regard to theoretically justified values or norms without facing the question why those affected do not problematize or attack such moral evils themselves (Honneth, 2007, p. 40).

From a dynamic perspective, it is important, for example, to investigate which socialization and politicization processes tend to precede environmentally harmful behavior, to what extent and in which contexts environment-related reflections become relevant to one’s own actions in the first place, and how these relate to certain internalized value structures, the image of one’s own past, and ideas of one’s own biographical and social future.

### Thesis 3: Environmental psychology should be seen in connection with social inequality and justice research

Environmental psychological issues should be addressed in conjunction with key global challenges. This means that especially social inequality research, poverty research, and the study of polarizations and divisions in societies have to be linked to environmental psychology, as well as the critical reflection of basic pillars of the contemporary world, such as the meaning of nationality and related limitations and possibilities of global action and public spaces of dialogue.

With strong ties to critical theory of the Frankfurt school, critical psychology particularly emphasizes the importance of social critique, and in this regard questions of power, context, or agency when looking at research contents and empowering approaches. Critical social psychologists (e.g., Tuffin, 2005) therefore point out that any contribution that relates individual action and experience to social contexts implicitly includes assumptions about society. The way in which any component of this society is described and explained is always also political, not least because it influences the self-image of its members. Directly connected to this are questions of justice as they are already clearly visible in the context of environmental psychology, for example, in the discourses on environmental justice and energy justice (see section “Alternative trends in current environmental psychology research fostering a critical perspective”). Perspectives of critical social psychology are crucial to follow processes of social transformation and to investigate how people deal with this situation and how this influences behavior, also on a group level (Kühn, 2015).

## Critical environmental psychology from a life course perspective

In this section, we use examples from our own research practice to illustrate how approaches from a life course-oriented critical social psychology can be applied to and linked to questions of environmental psychology. In doing so, we simultaneously aim to illustrate, with a more detailed example, how environmental psychology as a whole can benefit from an expanded focus such as we have outlined in the previous sections.

### A qualitative framework from a life-course perspective

Life course research combines two perspectives: On the one hand, it focuses on certain sections of specific biographies of individuals, for example, how entry into working life or the transition to retirement proceeds within study groups. On the other hand, the possibility spaces provided by social institutions are illuminated. This is based on the assumption that personal development trajectories can only be understood in the context of structurally shaped possibility spaces, such as those formed by the education and employment system. A life course-theoretical perspective stands out from approaches that speak rather vaguely of social community and associated culturally specific practices but do not further explore the significance of nation state and transnational regulatory systems. From a qualitative perspective, a particular focus is on the question of how and in what way different options to biographical trajectories are perceived and how this determines the everyday conduct of life. In the sense of critical research, this creates an approach to reconstructing mechanisms of constituting or reproducing social inequality structures. We can understand such qualitative life course research as a link between sociological and psychological approaches. Within the framework of psychology, there is a large overlap with cultural psychology, which, for example in the tradition of Bruner (1991; cf. Chakkarath, 2017), strives to illuminate subjective construction of meaning and its connection in the form of narratives. However, in life course research, a dynamic perspective on biographies and their social-structural, institutional anchoring is even more explicitly defined as the starting point of research.

Especially qualitative approaches are of high importance for critical research as they enable us to trace symbolic constructions of reality, which are the foundations for the actions of individuals and the formation of social groups. Kühn (2015) developed the qualitative approach of a *life-course oriented critical social psychology*, which can be characterized by three basic requirements:

(a) *everyday orientation*,
(b) *elaboration of biographical processes*, and
(c) *reconstruction of modes of perception of social structure*.

*(a) Everyday orientation*

Important environmental psychological insights can be gained by addressing basic pillars and constitutional principles of everyday life. This links directly to key questions of environmental psychology on how and why people in everyday life contribute to the destruction of global ecosystems without consciously intending to do so and might be very helpful to identify new starting points for effective pro-environmental actions in everyday life (cf. section “Introduction”).

**FIGURE 1:**
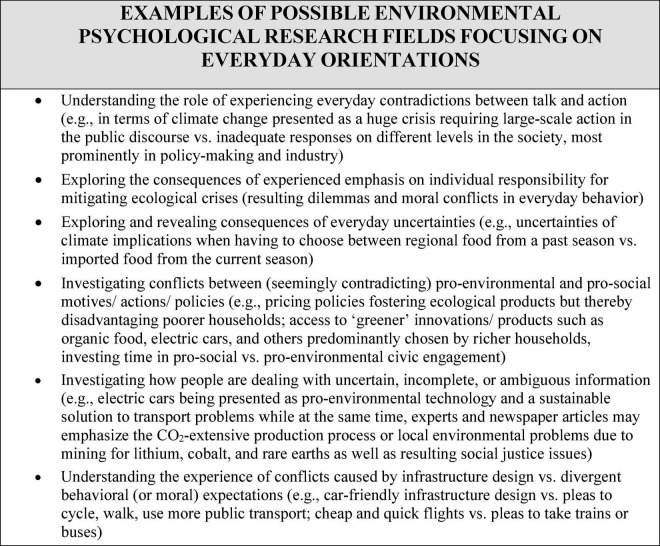
Examples of possible environmental psychological research fields focusing on everyday orientations.

A key advantage of qualitative everyday life-based research in environmental psychology is that it is able to capture ambivalences and contradictions. There are differentiated possibilities for tracing symbolic constructions of reality by individuals and groups by examining how people deal with uncertain, ambiguous, and contradictory initial conditions. We provide some examples of possible applications to questions from environmental psychology in Figure 1.

*(b) Analysis of biographic processes*

A major danger of primarily moment-based analyses is to view the lives of members of social groups predominantly as the consequence of individual decisions. A good example of this is the construct of free choice of occupation - in the sense that everyone is the architect of his or her own fortune and fate. Such a view ignores the fact that preceding family, school and occupational socialization processes are just as decisive for entering a profession as the institutional social-structural anchoring, shaping, and differentiation of certain occupational profiles, which were preceded by different historical development processes in different countries. Analogous considerations are also relevant for decisions that are the focus of environmental psychology, for instance regarding the use and purchase of means of transport in the context of research on mobility behavior.

From a biographical perspective, it can be reconstructed which conditions promote engagement in social movements and other forms of environmental activism (cf. section “Alternative trends in current environmental psychology research fostering a critical perspective”; see also Chawla, 1999; Matsuba and Pratt, 2013). The significance of the perception of justice issues in relation to social interaction as well as in connection with the environment can also be examined in connection with biographical planning processes and trajectories (cf. section “Alternative trends in current environmental psychology research fostering a critical perspective”).

**FIGURE 2:**
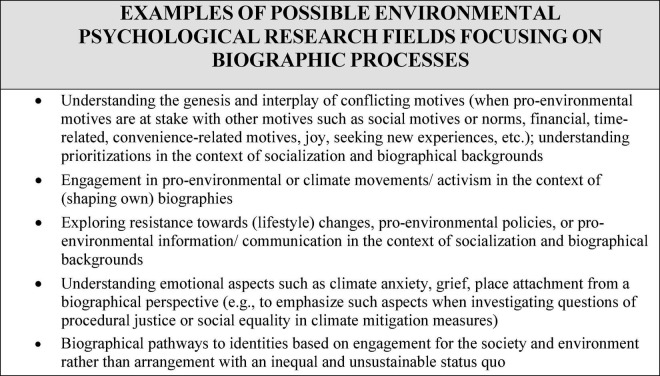
Examples of possible environmental psychological research fields focusing on biographic processes.

We provide some further examples of possible applications to questions from environmental psychology in Figure 2.

*(c) Reconstruction of modes of perception of social structure*

According to Kohli (1985, 1991), the life course can be understood as a social institution in the sense of a system of rules that structures central areas of life. By analyzing biographical processes and relating them to institutionally anchored structures, it is possible to understand how socialization processes lead to the formation of specific knowledge and value structures as well as habitualized ways of experiencing, perceiving, and acting, which ultimately form the basis for both self-limitations and the limited perception of possible options. Since individuals interpret the way people live together, interact with and feel connected to the environment in a specific way, it is important to make these patterns of interpretation the object of investigation by focusing on what images of social contexts and environment are drawn and for which social groups such images can be found and become meaningful. Exemplary questions could be: How do people explain social differences regarding the usage of alternative mobility options in societies, and which social groups are distinguished in this sense? To what extent is membership in different social groups constructed and what is this based on?

In section “Alternative trends in current environmental psychology research fostering a critical perspective,” we pointed out the growing importance of multi-level studies for environmental psychology. This life course theoretical approach fits in with this, taking a microscopic view of subjective perceptions and biographical trajectories, but at the same time linking them to social institutions and structural conditions on a meso or macro level.

**FIGURE 3:**
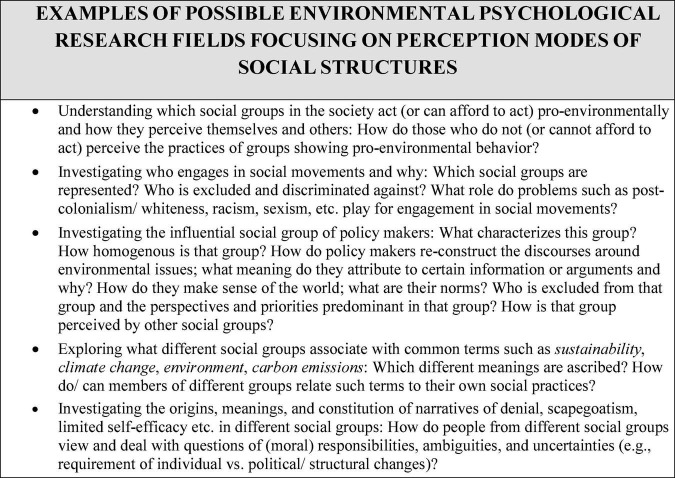
Examples of possible environmental psychological research fields focusing on perception modes of social structures.

We provide some more examples of possible applications to questions from environmental psychology in Figure 3.

The conceptual approaches developed with the help of such a life-course oriented perspective are also relevant for environmental psychology. In the following, we will elaborate on this by drawing on examples from two of our own research projects.

The first project was about biographical trajectories in different training occupations. The project was part of a large-scale *DFG Collaborative Research Centre* that focused on the significance of status passages and risk positions in the life course. The surveys in the subproject *Status Passages into Employment* were carried out as a prospective longitudinal study in the late 1980s and 1990s, headed by Walter R. Heinz (Heinz et al., 1998; Kühn and Witzel, 2000; Schaeper et al., 2000; Heinz and Krüger, 2001)^4^. Kühn’s research focused on how young, still childless adults dealt with the biographical option of starting a family and how this influenced biographical decisions, also in the professional sphere (Kühn, 2004). In accordance with a critical social psychological perspective, the project also dealt with unequal opportunity structures in different occupational environments and their significance for subjective modes of perception and action. Conceptually, we will present two models developed in the project in the following that we consider to be highly relevant for environmental psychological issues in section “Biographical Planning and Ambivalences”: the model of *biographical planning* as well as a conceptual distinction for dealing with *ambivalence* in everyday life.

The second, more recent project dealt with identity constructions in the life course during the first year of the COVID-19 pandemic. During the project, qualitative interviews were conducted in Germany, Austria, and Brazil. The example we will provide in section “Changing meanings of consumption as identity work during the COVID-19 pandemic” refers to the Brazilian sub-study, for which more than 50 problem-centered interviews were conducted between April and May 2020 with Brazilians from different social groups (Kühn et al., 2020). Kühn et al. (2020) examined the significance of consumption for one’s own identity work in times of social distancing. In this article, we will emphasize the results that show how the experience of the pandemic can contribute to a changed understanding of oneself and of consumption.

### Biographical planning and ambivalences

There are considerable differences in the way young childless adults with a desire to have children deal with the option of starting a family. As part of the study *Status Passages into Employment*, young adults who had completed vocational training in various occupations were interviewed a total of three times at intervals of 2–3 years using qualitative guided *problem-centered interviews* (Witzel and Reiter, 2012) about their previous biographical trajectories, their current life situation, and their future life trajectories.

Critical social psychological research requires a concept of planning that is able to capture different forms of subjective engagement with biographical options without normatively tying them to particular notions of competence or rationality. Based on *Grounded Theory* (Strauss and Corbin, 1990; Witzel, 1996; Kelle, 2007) by the comparison of qualitative interviews, Kühn (2001, 2003, 2004, 2020b) developed the concept of *biographical planning*, which is particularly well suited to differentially capture the way individuals deal with structurally conditioned individual ambivalences and biographical uncertainty from an empirical perspective. The approach of biographical planning investigates which biographical goals actors develop and in which way individuals thematize biographical options, relate them to desired goals, and anticipate ways and activities to achieve these goals.

According to the developed typology, ideas oriented toward one’s own biographical future differ, on the one hand, in how certain areas of life are linked as well as how the life courses of significant others are included (dimension *Interlinking*), how broad and clear the horizon of one’s own ideas about the future is (dimension *Horizon*), and how ideas about the future develop, e.g., whether they remain constant over a period of time, fluctuate, etc. (dimension *Development*).

**FIGURE 4:**
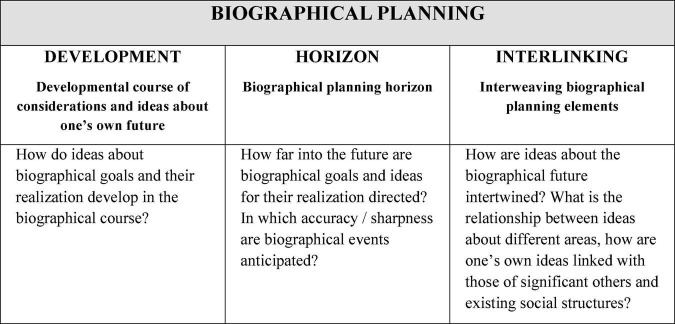
The concept of biographical planning (adapted from Kühn, 2004).

**FIGURE 5:**
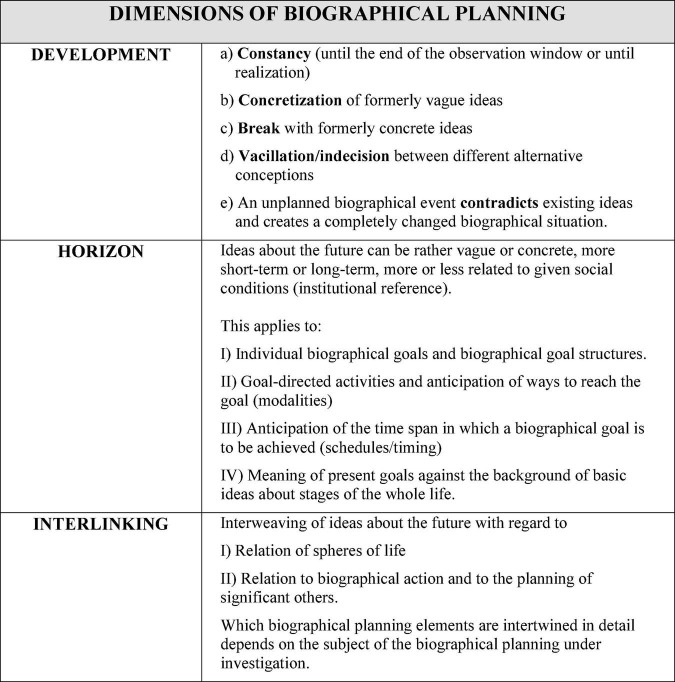
Elaborations on the three biographical planning dimensions (adapted from Kühn, 2004).

Figures 4, 5 describe the concept in detail.

Such an approach of biographical planning can be used for different environmental psychological questions that are related to biographical decisions. For example, planning processes for larger investments, e.g., in the fields of mobility or energy supply, could be linked to other areas of life and it could be differentiated which different planning modes exist and how they are related to different contextual conditions. It could also be examined how perceptions of climate crisis play a role on biographical planning processes for family formation (e.g., Schneider-Mayerson and Leong, 2020).

**FIGURE 6:**
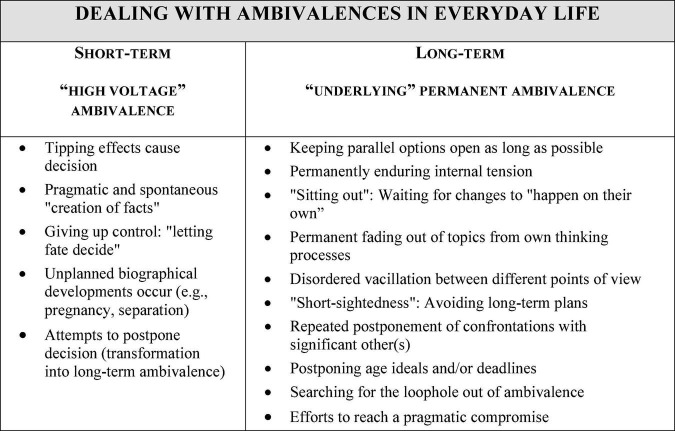
Differences in dealing with short-term vs. long-term experiences of ambivalence in everyday life (adapted from Kühn, 2004).

In the study *Status Passages into Employment* it became clear how significant ambivalent initial situations were for the everyday life and biographical course of young adults. Especially in view of structural obstacles to reconcile a successful professional career with high normative demands on one’s own role as a parent, this created a tense initial situation for many childless persons, which at the same time represented a significant obstacle to long-term biographical planning processes. Once again, a heuristic model was developed from the comparison of the interviews on the basis of grounded theory, which distinguishes between (a) a permanent ambivalence existing over a longer biographical period and (b) an ambivalence experienced as highly tense with pressure to make decisions. The model differentiates between various biographical ways of dealing with these types of ambivalences (Kühn, 2004; see figure 6).

From the perspective of environmental psychology, structurally determined ambivalences are also important, for example with regard to ecological, economic and social aspects of sustainability as guidelines for decisions at the management level in organizations, but also with regard to possible expensive purchases or the decision to change one’s own diet. Here, such a model and the associated knowledge about different biographical ways of dealing with things offer starting points for further research.

### Changing meanings of consumption as identity work during the COVID-19 pandemic

From a life course perspective, the construction of identity has to be understood as an ongoing development process linked to one’s own biography, which takes place as identity work in confrontation with the social world (e.g., Rosa, 1998; Keupp et al., 2002; Ehnis et al., 2015; Kühn, 2015, 2020a; Kühn et al., 2020). In line with this understanding, we can understand identity always as a specific subjective positioning that connects the experience of the present with interpretations of the biographical past and imagined future. The reconstruction of identity work may not be limited to the analysis of verbally articulated self-images and self-assessments of one’s own person but has to focus on everyday life.

Especially in linking with approaches of the German sociologist Rosa (1998) and the social psychology group of Keupp et al. (2002), Kühn (2020a) proposed to differentiate identity work on three levels, summarized in the *normative ABC model of identity* (Kühn et al., 2020):

(A) Striving for *authenticity* and coherence: appreciating oneself, experiencing oneself as coherent and genuine, coming to terms with oneself.
(B) Striving for *belonging* and recognition: to experience oneself as integrated in society and as a valued part of a community.
(C) Striving for *control* and responsibility: being able to experience, shape and act effectively.

According to this model, identity work is a life long task, understanding identity as a dynamic construction that has to be re-established again and again in the course of life. The normative expectation is to constitute oneself in a specific way on three levels throughout life: to understand oneself as a coherent and unique person (authenticity), to understand oneself as a member of different groups (belonging), to make one’s own decisions and in this way to exert a controlling influence on life (control). Identity work is It is connected with the effort to understand oneself and the world.

Within the research project *Identity constructions during the pandemics* (cf. section “A qualitative framework from a life-course perspective”), this normative ABC model of identity (Authenticity, Belonging, Control) forms a basis for investigating the symbolic significance of consumption for identity constructions.

The identity perspective has been used as theoretical ground to analyze and explore the symbolic meanings that individuals give to consumption and to understand how these meanings have changed during the course of the COVID pandemic. Kühn et al. (2020) compared the interviews in terms of references to the importance of consumption for the construction of identity on the ground of a thematic analysis (Braun and Clarke, 2006), following a reflexive basic understanding (Braun and Clarke, 2019). The results showed a two-faced picture. On the one hand, consumption contributed to the reproduction of social inequality and even lead to polarizations within the Brazilian society becoming more significant. The researchers observed a reinforcement of social inequalities related to consumption, but also regarding the inclusion in the work sphere:

Whereas for many rather poor people in Brazil, the possibility to work even within the pandemic is a matter of survival, as they need the income to buy food, for richer people there is scope and opportunity to reflect on one’s own role in society free from existential constraints. These findings show the extent to which normalcy continues to be unjust during and after the pandemic (Kühn et al., 2020, p. 809).

**FIGURE 7:**
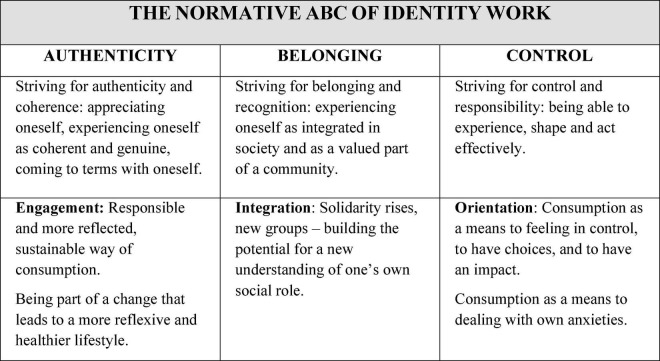
Analyzing the importance of consumption for the construction of identity during the COVID-19 pandemic in Brazil with the normative ABC model of identity (adapted from Kühn et al., 2020).

On the other hand, Kühn et al. (2020) were able to analyze that, during the pandemic, people also reflected on their consumption and made efforts to change their own consumption behavior. Furthermore, consumption could also contribute to providing orientation, to feeling like an integrated member of a community, and to strengthening one’s own commitment. Figure 7 provides an overview of these findings.

Kühn et al. (2020) see hopeful signs in their findings from a critical social psychological perspective, an assessment that is also relevant for environmental psychology:

These include the emphasis on the responsibility as a consumer for sustainable social development as well as the experience of solidarity and the formation of new identities, which could form the basis for future political endeavors. Different modes of consumption are possible, e.g., a reflexive consumption of regional products in order to strengthen and promote regional traders, from a social identity perspective also in order to feel part of the community, not feeling isolated, but as a productive piece of a whole (Kühn et al., 2020, p. 808).

The example of the changing meaning of consumption during the pandemic shows how fruitful it can be also for other areas of environmental psychology to address them from a life-course identity perspective, such as regarding the meaning of a vegan diet, the study of biographical turning points, and related decisions to change one’s actions and to engage in social movements.

## Conclusion

Environmental psychology has a long tradition and is a recognized research discipline that is also valued in practice. In view of climate change and discussions about a sustainable design of social transformation processes, for example, in connection with the digitalization of everyday worlds, the importance of research in environmental psychology is becoming increasingly apparent to wider circles. It seems all the more important to us that environmental psychology opens itself even more to exchange and discourse with representatives and approaches of other disciplines and that promising joint projects emerge from the contact that link individual behavior with reflections on social development.

In this respect, many promising projects in environmental psychology can already be identified, some of which we have listed during the course of this paper. At the same time, given the complexity of environmental psychology issues, we see a certain danger of fragmentation that could lead both to promising projects not getting the attention they deserve and to more work being done side by side rather than together.

In this respect, a shared self-understanding in terms of a critical environmental psychology would be helpful. We have developed points of reference for this in the article, which we take to be a departure into discourse rather than the end point of such discussions. In our understanding, a critical approach is characterized in particular by its reflexivity, dynamic understanding, and questioning of power and inequality structures as a milestone on the way to a more just and sustainable world. As an example of a critical understanding in this sense, we have discussed the possibilities of a qualitative life course approach.

In this sense, environmental psychology would be more concerned with strengthening a socialization perspective that focuses on identity work in confrontation with social structures and thus, in particular, allows conclusions to be drawn about how partly contradictory, ambivalent, or ambiguous normative social expectations are reflected in everyday life and unequal biographical life paths. Qualitative research in particular is suited to show, from a critical perspective, how habitualized practices, symbolic, and narrative constructions of reality are linked to social inequality as well as suboptimal behavior patterns from an environmental sustainability perspective.

Conceptually, the approach of the normative ABC of identity work, biographical planning, and the distinction between two biographically relevant forms of ambivalence exemplified how such research can be made fruitful for central environmental psychological questions. This preliminary conceptual work provides a foundation that can be used for numerous future projects.

We make no claim to completeness with this article, but see it as an impulse. In keeping with our reflexive understanding, we believe it is important that environmental psychologists also reflect on what constitutes its own discipline and how research in the field can be meaningfully expanded and supplemented.

Following on from this, we would like to issue a “call for action”: We would be delighted if colleagues expressed interest in developing a comprehensive critical research agenda and further contributing to building a global research network and critical community together.

Especially in view of numerous global challenges such as climate change and divisions within and between societies that shape our contemporary everyday life, we consider it one of the most important tasks of environmental psychology to make contributions that deal with transformation and can contribute to shaping sustainable social and structural change and clearly identify associated dangers.

## Author contributions

TK and SB contributed to the conceptualization of the manuscript, wrote sections of the manuscript, reviewed and edited the original draft, and approved the submitted versions. TK was responsible for funding acquisition and supervision.

## Footnotes

1 While acknowledging diverse conceptualizations of the terms *sustainability* and *sustainable development*, in this article, we refer to the comprehensive concept defined by the World Commission on Environment and Development (1987). This concept encompasses economic, ecological, and social aspects and aims at ensuring to meet the needs of all humans (hereby emphasizing the needs of poor and marginalized people) in the present without harming the needs of all humans in future generations.

2 This debate is usually labeled as a conflict between *intent*- vs. *impact*-oriented research (cf. Moser and Kleinhückelkotten, 2018): Intent-oriented research in environmental psychology focuses on peoples’ pro-environmental motivation and moral norms, mainly in the sphere of habitualized household behaviors. Studies show that such factors can indeed predict the readiness to act pro-environmentally, however, the environmental impact of performing such behaviors was found to be relatively low. Impact-oriented research promotes to focus on spheres of behavior with a relatively high environmental impact first, such as energy-related investment decisions. Studies have found that socioeconomic variables are much more important predictors here than in the sphere of habitualized household behaviors. As environmental psychologists have traditionally been leaning more toward studying pro-environmental behavior from an intent-oriented perspective, impact-oriented researchers suggest more studies on predictors of high-impact behaviors and to investigate closer how the difference in the most influential predictors can be explained. The long-ongoing debate within the environmental psychology community has recently gained new momentum (e.g., Nielsen et al., 2021; van Valkengoed et al., 2021).

3 Researchers from the field of environmental psychology have recently linked the term *critical environmental psychology* particularly to critical psychology in the tradition of Holzkamp (1983; cf. Krenzer and Kreil, 2019, pp. 163-164; Räthzel and Uzzell, 2019, pp. 1376-1377). We completely agree with the benefits that would come from integrating insights from this research tradition systematically into research practices and theories in environmental psychology. However, in this article, we use the term as an umbrella term, referring to a general approach rather than a specific theoretical framework. Such an approach would be characterized by critically (self-)reflecting upon the underlying paradigms, theories, conceptualizations, and methods of research in the field by emphasizing the importance of social relations, social inequalities and (power) structures as well as by taking historical-cultural contexts, biographical socialization processes, and the dynamics of developments within and between individuals and society into account (see section “Fundamental considerations on the importance of critical social psychology for environmental psychology”; cf. Tuffin, 2005; Parker, 2007; Billig, 2008; Kühn, 2015).

4 In total, surveys were conducted in four waves at intervals of approximately three years between 1989 and 1997. In the fourth survey wave, only a standardized survey was conducted. problem-centered interviews with *n* = 91 respondents are available from the first three waves, who were interviewed at three points in time.
